# Factors to Improve the Management of Hepatitis C in Drug Users: An Observational Study in an Addiction Centre

**DOI:** 10.1155/2010/261472

**Published:** 2010-07-18

**Authors:** Joseph Moussalli, Helene Delaquaize, Dominique Boubilley, Jean Pierre Lhomme, Jules Merleau Ponty, David Sabot, Anne Kerever, Marc Valleur, Thierry Poynard

**Affiliations:** ^1^Service d'hépatologie, Université Pierre et Marie Curie Liver Center, Hôpital La Pitié Salpêtrière, 75013 Paris, France; ^2^Hôpital Marmottan, General Medicin, 17 rue d'Armaillé, 75017 Paris, France

## Abstract

Barriers to management of HCV in injection drug users are related to patients, health providers, and facilities. In a primary care drug user's addiction centre we studied access to HCV standard of care before and after using an onsite total care concept provided by a multidisciplinary team and noninvasive liver fibrosis evaluation. A total of 586 patients were seen between 2002 and 2004. The majority, 417 patients, were HCV positive and of these patients 337 were tested positive for HCV RNA. In 2002, patients were sent to the hospital. with the Starting of 2003, patients were offered standard of care HCV management in the center by a team of general practitioners, a consultant hepatologist, psychiatrists, nurses, and a health counsellor. Liver fibrosis was assessed by a non invasive method. In 2002, 6 patients had liver fibrosis assessment at hospital facilities, 4 patients were assessed with liver biopsy and 2 patients with Fibrotest-Actitest. 2 patients were treated for HCV at hospital. In 2003 and 2004, 224 patients were assessed with Fibrotest-Actitest on site. Of these, 85 were treated for HCV. SVR was achieved in 43%. We conclude that the combination of an onsite multidisciplinary team with the use of a noninvasive assessment method led to improved management of HCV infection in drug users' primary care facility.

## 1. Introduction

Hepatitis C is a major issue with intravenous drugs users (IVDUs), where the prevalence of HCV is 50% to 80% [1, 2]. In addition, the prevalence of HCV infection remains high (40% to 60%) despite harm reduction programs targeted to recent IVDU [1]. It has been estimated that approximately 5000 new HCV cases occur per year in France of which 70% are related to drug use. The incidence of HCV in IVDU is 10/100 persons, years versus 0.65/100.000 persons, years in blood donors [1]. IVDUs constitute the principal transmission reservoir for HCV. HCV infected individuals are at risk of developing cirrhosis, end stage liver disease, and liver cancer [3]. Nearly 50% of IVDU are infected with more easily treatable HCV genotypes 3 or 2 [4].

 IVDUs are evaluated less frequently by medical personnel and treated less than other patients despite high willingness to receive therapy [5]. In the United States, Canada, and Australia, only 1%–6% of current and former IVDUs have received HCV treatment [6–9]. The reasons for exclusion from treatment are not based on evidence from the literature. Physicians fear psychiatric side effects, bad adherence to HCV treatment, and reinfection [10]. The high probability of residual drug abuse or alcohol consumption by these patients often is invoked to exclude them from treatment. Despite those considerations, several successful HCV treatment studies involving illicit drug users have been published over the past years [11–26]. 

We tested the hypothesis that evaluation and treatment of HCV infected IVDU could be possible after implementation of relevant personnel and tools in a primary care facility. In a drug users' addiction centre with a very low HCV treatment uptake, we conducted an observational study of HCV standard of care management after implementation of an onsite multidisciplinary team and noninvasive fibrosis assessment.

## 2. Methods

### 2.1. Patients

The cohort comprised patients attending the centre between January 2002 and December 2004. The centre offered addiction services such as buprenorphine or methadone therapy, needle exchange, counselling, and prevention. Other routine services included general primary care, nursing, and social support. Among 586 patients seen in the centre at that time, 417 (70%) and tested HCV positive. Of these, 337 were tested HCV RNA positive by PCR.

### 2.2. Methods

In 2002, HCV patients were referred to hospital. Beginning in 2003, the new strategy, including an on-site multidisciplinary team and non-invasive assessment was implemented. Patients were no more referred to hospital for HCV. The onsite multidisciplinary team was composed of five general practitioners qualified in addictive medicine, a hepatologist on loan from a hospital, psychiatrists, two nurses, a health counsellor, and a secretary. The general practitioners, who provided a permanent presence in the center, served the pivotal role of building a quality long-term relationship with the patients by treating addiction and Hepatitis C simultaneously. The team benefited from four hours of training per month aimed at managing the HCV infection. The hepatologist's mission was to motivate, trainees and coach the general practitioners. The interaction between the hepatologist and the general practitioners was facilitated through regular meetings where each patient's file was reviewed, discussed with the entire team, and was used as a means of training. The indications for treatment were decided during these meetings. In addition to these meetings, the hepatologist was easily accessible by phone to answer the team's questions and to solve problems within his realm of competence. When necessary, the psychiatrists completed an initial evaluation and ensured the follow-up, in some cases very closely. The nurses' role was to motivate patients, provide them with therapeutic education, initiate the treatment, provide ongoing access for patients' weekly injections of Pegylated interferon, when needed, as well as to monitor patients' adherence to the treatment. The nurses provided permanence and were immediately available during the center's working hours. The health counselor provided social health service, informing and educating patients and spending a considerable amount of time discussing and listening to the patients' needs.

The patient's clinical and psychiatric evaluation was completed at the center. Blood tests were performed in a centralized manner in the same laboratory. 

According to official recommendations, HCV treatment was indicated in patients with a fibrosis score ≥F2 given their motivation and psychiatric and socioprofessional situations, which arose as the result of a previous course of consultations with the general practitioner during an average of six months. Patients were not referred to hospitals.

The two periods 2002 and 2003-2004 were compared in terms of disease evaluation and initiation of treatment. The two groups of patients were also compared in terms of age, drug consumption, and opiate substitution treatment.

### 2.3. Statistical Methods

The *χ* ^2^ test was used to analyse the qualitative variables and variance analysis was used for the quantitative variables. The threshold of significance retained was 5%. The calculations were carried out with NCSS software.

## 3. Results

**Figure 1:**
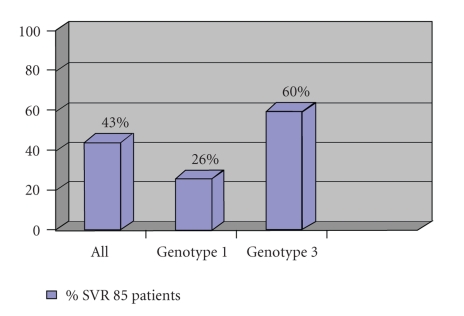
SVR in intention to treat analysis.

In 2002, 6 patients underwent fibrosis evaluation. 4 patients had liver biopsy and 2 patients had Fibrotest-Actitest noninvasive testing in hospital. 2 patients were treated for HCV in hospital settings. Between January 2003 and December 2004, 224 patients (group 1) underwent complete evaluation in the centre including Fibrotest-Actitest, while 113 patients (group 2) did not. Among the patients of group 2, only four patients out of 113 (3.5%) had been already evaluated by liver biopsy at hospital and only two patients (2%) had been treated in 2002. Amongst group 1, 85 patients (38%) were treated onsite for HCV in the same period. Comparison of treatment uptake between the 2002 and 2003-2004 periods was highly significant in favour of the latest (38% versus 2% *P* < .001). Comparison of clinical and biological characteristics of groups 1 and 2 is given in table 1. In group 2, there was a lower rate of opiate substitution (55% versus 76% *P* < .001) and a higher rate of drug abuse (61% versus 17% *P* < .001). In group 1, 85 patients (38%) were treated between January 2003 and September 2004. Among the patients of group 1, the average index of fibrosis was 0.46 ± 0.26 (F1-F2), the average index of activity was 0.46 ± 0.25 (A1-A2). Eighty-one patients (36%) had minimal fibrosis F0, F0-F1, and F1; 66 patients (30%) had moderate fibrosis F1-F2 and F2; 77 patients (34%) had severe fibrosis F3, F3-F4, and F4. The average age of treated patients was 42 ± 5.2 years. The average index of fibrosis was 0.62 ± 0.19 (F3) among treated patients versus 0.39 ± 0.26 (F1-F2) among untreated patients (*P* < .001). Among the treated patients, 20 (23%) used drugs, 37 (44%) had alcohol consumption higher than 50 grams per day, and 62 (73%) had opiate substitution treatment. In intention to treat 37 patients (43%) had a sustained response, 10 among 38 genotype 1 (26%) and 25 among 42 genotype 2 and 3 (60%) patients (Figure 1). Fifteen patients (17%) stopped the treatment prematurely: four patients were lost to follow-up, one patient had a cardiac complication, one patient had hyperthyroidism, five patients suffered from adverse psychiatric events in the form of depression and/or severe alcoholism, and four patients stopped treatment after the first injection because of the anxiety generated by a pseudo syndrome of opiate withdrawal.

## 4. Discussion

While HCV appears to be a major prevalent issue in IVDU patients, it is clearly undertreated in this population. On the other hand, IV drug use is the first risk factor of HCV transmission and risk reduction policies did not reduce dramatically HCV transmission and prevalence in this population. HCV is associated with a risk of morbidity and mortality. Combination treatment consisting of pegylated interferon and ribavirin has been shown to be highly effective, achieving viral clearance rates between 55% and 85% depending on genotype [27, 28]. 

Decreased uptake of treatment among IVDUs is probably attributed to both healthcare provider and patient-associated factors. Treatment for HCV infection among IVDUs may be withheld by physicians to perceived “difficult” patients based on concerns of adherence and treatment sideeffects [10]. Current hospital protocols seem of limited utility for treatment of HCV in IVDUs [10]. On the patient's side psychiatric comorbidities and poor social support are common among IVDUs and HCV treatment may not be a high priority for them. Therefore, new strategies are required to treat HCV in this population. 

It has been demonstrated that when specific programs are developed, IVDU can be successfully treated for HCV. Response rates following HCV treatment in IVDU [11–26] are close to the response rates in large clinical trials [27, 28]. 

The aim of this study was to observe the contribution of a different healthcare organisation on HCV standard of care evaluation and treatment. Within a primary healthcare setting for IVDU, HCV-positive patients were able to benefit from a strategy that consisted of an onsite total care concept provided by a multidisciplinary team including a referred delegated hepatologist and HCV evaluation using Fibrotest-Actitest a biomarker noninvasive method. We did not use combination with Fibroscan because it was not easily accessible as a routine assessment method at that time. Among a group of 417 HCV-positive patients of which 337 subjects were viremic, 224 were evaluated for fibrosis and 85 of them were treated after two years. During the year which preceded the realization of this “all under one roof total care concept”, only 6 liver evaluations and 2 treatments were carried out at hospitals. Patients undergoing complete evaluation had a significant higher rate of opiate substitution treatment. Substitution appeared to be a major precondition for access to care in this population.

Treatment indications followed official guidelines recommending HCV treatment in patients with fibrosis ≥F2. The rate of sustained viral response was 43% in intention to treat analysis. This result is reasonable if we consider that the treated patients had an average F3 fibrosis index and that 50% had genotype 1 infection. No serious adverse psychiatric event was observed. 

HCV screening, assessment, and treatment seem feasible and efficient in IVDU. It requires a specific adaptation of the healthcare system to this particular population. In our study, it appears more efficient to propose onsite multidisciplinary care in an IVDU primary care centre rather than referral to hospital.

## Figures and Tables

**Table 1:** Comparison of clinical and biological characteristics between groups 1 and 2.

	Group 1 *N* = 224	Group 2 *N* = 113	
Mean age	40	37	
Genotype 2 or 3	46%	49%	
Drug use	38 (17%)	69 (61%)	*P* < .001
Alcohol use	83 (37%)	45 (40%)	NS
Substitution	169 (76%)	62 (55%)	*P* < .001
HCV treatment	85 (38%) (2003-2004)	2 (2%) (2002)	*P* < .001
